# Implementing innovative financing mechanisms in global health – perspectives of stakeholders from global health organisations: a qualitative study

**DOI:** 10.7189/jogh.16.04260

**Published:** 2026-09-25

**Authors:** Sze Wan Ching, Marius Wamsiedel, Jinkou Zhao

**Affiliations:** 1 Global health research center, Duke Kunshan University, Kunshan, China; 2 Faculty of Sociology and Social Work, University of Bucharest, București, Romania; 3 Institute of Global Health, University of Geneva, Geneva, Switzerland; 4 Fudan Institute of Advanced Studies in Global Health, Fudan University, Shanghai, China

**Keywords:** global health, health financing, innovative financing, qualitative research, global health organisations

## Abstract

**Background:**

The COVID-19 pandemic and recent geopolitical shifts have strained traditional funding for global health, increasing the interest in innovative financing mechanisms. We explore how international organisations perceive and implement these mechanisms as complements to traditional funding.

**Methods:**

In this qualitative study, we conducted semi-structured interviews with eleven senior experts from major global health organisations, gathered *via* purposive and snowball sampling between May and July 2024. We analysed data using systematic coding and thematic analysis, with trustworthiness enhanced through member checking and peer debriefing.

**Results:**

Participants revealed that innovative financing mechanisms are not replacements, but strategic complements to traditional aid. We identified three primary patterns in our data: an evolution from direct funding to complex, multi-stakeholder risk-sharing arrangements; a fundamental transformation of stakeholder relationships, increasing coordination complexity; and the critical role of institutional capacity in successful implementation. Analysis of five innovative financing mechanisms, namely Advanced Market Commitments, Blended Finance, the International Finance Facility for Immunization (IFFIm), Debt2Health, and Micro-levies, demonstrated distinct patterns in complexity, coordination, and risk distribution across said mechanisms. The success of the implementation depended on clear problem definition, stakeholder alignment, and robust risk management. Blended Finance and IFFIm showed the highest complexity, while Debt2Health offered a more streamlined, triangular model.

**Conclusions:**

The effectiveness of innovative financing mechanisms is heavily dependent on existing institutional frameworks and local context. They represent an evolution in health financing, offering tools for resource mobilisation and efficiency, but their success hinges on careful attention to implementation context, stakeholder coordination, and institutional capacity. Policymakers should focus on matching mechanisms to specific contexts and building coordination capabilities alongside technical innovation.

While the COVID-19 pandemic brought global health to the forefront of political discourse, it paradoxically strained health financing. The massive redirection of domestic resources toward pandemic response, coupled with pandemic-induced economic downturns in donor countries, diverted attention and funding away from established global health priorities such as HIV, tuberculosis, and malaria, exposing the fragility of traditional funding models [1,2]. Here, we use ‘traditional financing’ to refer to any direct bilateral and multilateral official development assistance. This includes government-to-government aid, contributions to multilateral organisations like the World Bank or the World Health Organization (WHO), and grants from philanthropic foundations such as the Global Fund and the Bill & Melinda Gates Foundation. These mechanisms are characterised by their grant-based nature, reliance on donor government appropriations and annual budget cycles, and often, predefined allocation criteria tied to specific projects or disease programmes [3]. This situation has renewed interest in innovative financing mechanisms, which we define as approaches beyond traditional aid that either raise new funds (*e.g.* through micro-levies or debt swaps) or improve the efficiency and impact of existing funds (*e.g.* through blended finance or results-based funding) [4].

Innovative financing mechanisms have emerged as potential solutions to address funding gaps and inefficiencies. Mechanisms like the International Finance Facility for Immunization (IFFIm) transform long-term donor pledges into immediate resources through bond markets [5], while Debt2Health converts debt obligations into health investments [6]. Blended Finance combines public and private capital to leverage development assistance [7]. However, existing literature highlights risks, including high setup costs, volatility of non-bilateral funding, and questions about sustainability and scale [8]. While the potential benefits of innovative financing mechanisms are recognised, there remains a limited understanding of how they are operationalised as complementary approaches to traditional funding from the perspective of global health organisations.

In this exploratory study, we investigate how key stakeholders in major global health organisations perceive, evaluate, and implement innovative financing mechanisms. We focus on understanding their practical experiences, the challenges they faced, and their perceptions of the potential of various mechanisms in addressing current global health funding gaps.

## METHODS

In this qualitative study, we wanted to capture in-depth perspectives on the complex implementation of innovative financing mechanisms. We identified participants through purposive and snowball sampling, targeting senior professionals from global health organisations involved in funding allocation and implementation of innovative financing mechanisms. While governmental and recipient country perspectives are important, we prioritised global health organisations, as they directly engage with both traditional and innovative financing. In this sense, we specifically focused on the Global Fund, Gavi, Unitaid, and the World Bank, as they represent the primary multilateral entities engaged in designing, operationalising, and scaling innovative financing for global health. These organisations are not merely participants in the field, but are the primary entities responsible for the design, piloting, and scaling of the mechanisms under study (*e.g.* IFFIm, Advanced Market Commitments, and Debt2Health). Together, they oversee a substantial portion of innovative financing instruments, making their perspectives uniquely informative. Consequently, they provide the most robust and relevant primary data sources for understanding the transition from traditional to innovative models.

### Data analysis

We collected data between May and July 2024 *via* semi-structured in-person or online interviews which lasted 45–60 minutes each. An interview guide (Appendix A in the **Online Supplementary Document**) focused the discussion on experiences with traditional and innovative financing, including challenges and opportunities. Interviews were audio-recorded, transcribed verbatim, and anonymised. Each participant provided oral consent during the recorded interview, and was offered the option to opt out from participation at any time during the interview.

We determined our sample size by the principle of thematic saturation, the point at which additional interviews no longer yielded new insights or themes [9]. Data collection continued until no novel information emerged from interviews, indicating that saturation had been achieved. In qualitative research of this nature, the depth and expertise of participants are often prioritised over numerical breadth to capture nuanced systemic insights. While governmental and recipient country perspectives are undoubtedly important, we intentionally prioritised global health organisations, as they serve as the primary intermediaries and architects of these financing mechanisms.

Our data analysis followed a systematic coding process involving initial line-by-line coding; focused coding to consolidate codes into categories; and the development of themes, where categories were organized into overarching themes addressing the research questions. Analytical memos were maintained throughout the interviews.

Trustworthiness was ensured through member checking, peer debriefing with senior researchers, maintenance of an audit trail, and contextual description to support transferability. Specifically, we returned emerging interpretations to the participants so that they could verify the accuracy of perspectives. We engaged senior researchers to test analytical assumptions. We also kept a meticulously documented chronological record of the entire research process, including raw data files, field notes, codebook iterations, analytical memos, and decisions made regarding theoretical directions. We conducted the analysis in NVivo, version 14 (QSR International, Burlington, Massachusetts, USA).

## RESULTS

The final sample consisted of eleven participants from the Global Fund, Gavi, Unitaid, and the World Bank (**Table 1**). All participants held senior positions in their respective organisations and had professional experience ranging from 8 to 15 years in global health financing. Their educational backgrounds included advanced degrees in public health, economics, and international development, providing a strong theoretical foundation for their practical expertise. This combination of academic training and substantial professional experience enabled participants to offer both strategic and operational insights into innovative financing mechanisms.

**Table 1:** Profile of study participants (n = 11)

ID	Organisation	Department/role	Expertise areas	Sampling method
P1	The Global Fund	Grant management division	Co-financing, sustainability	Purposive sampling
P2	The Global Fund	Programmatic monitoring department	Epidemiology	Purposive sampling
P3	The Global Fund	Specialist blended and health finance	Blended and health finance	Snowball sampling
P4	The Global Fund	Health finance department	Health finance	Purposive sampling
P5	The Global Fund	Private sector engagement department	Health finance, private sector engagement	Snowball sampling
P6	Gavi	Senior manager for resource mobilisation	Resource mobilisation	Purposive sampling
P7	Unitaid	Senior resource mobilisation manager	Resource mobilisation	Snowball sampling
P8	Unitaid	Corporate performance and impact results	Resource mobilisation	Purposive sampling
P9	The Global Fund	Health finance department	Debt2Health	Snowball sampling
P10	World Bank	Senior economist	Health, nutrition, and population	Snowball sampling
P11	The Global Fund	Health finance department	Resource mobilisation	Purposive sampling

### Implementation experiences with key innovative financing mechanisms

The participants detailed distinct experiences with five major innovative financing mechanisms, highlighting their unique features and implementation challenges.

#### Advanced Market Commitments

Advanced Market Commitments are designed to incentivise vaccine development and manufacturing for diseases predominantly affecting low-income countries. Donors can use them to legally commit funds to guarantee a viable market for future vaccines, encouraging pharmaceutical companies to invest in research and production. Gavi’s pneumococcal Advanced Market Commitment, launched in 2009, represents the most prominent example, successfully accelerating access to pneumococcal vaccines in dozens of countries. However, participants noted that Advanced Market Commitments faced challenges in securing sustained political commitment across budget cycles. One participant noted: ‘Securing funding across multiple budget cycles is often complicated by shifting economic conditions’. High transaction costs (approximately 7% administrative overhead *vs*. 3% for direct funding) and significant context dependency on local manufacturing and supply chain capacity were also critical barriers.

#### Blended Finance

Blended finance strategically uses public or philanthropic capital to mobilise private sector investment for development objectives. In global health, this typically involves development finance institutions, global health organisations, and private investors co-investing in health infrastructure or service delivery, with public funds absorbing higher risks to attract private capital. Implementation revealed profound coordination challenges. A participant emphasised institutional misalignment: ‘Different organisations have their own processes... the way how they do the audit, the way they disburse the money’. Case studies, such as the Global Fund facilitating concessional loans in Benin (USD 50 million) and Togo (USD 25–30 million), demonstrated potential but required extensive technical assistance. Participants described blended finance as ‘not well-organised or standardised’, with time-consuming, risk-averse approval processes.

#### IFFIm

The IFFIm is a multilateral financing instrument that accelerates the availability of funds for Gavi’s immunisation programmes. It converts long-term, legally binding donor government pledges into immediately available cash resources by issuing bonds in international capital markets. The World Bank serves as IFFIm’s treasury manager, providing institutional credibility and financial expertise. The IFFIm was highlighted as a successful market-based mechanism. Its strength lay in strong market acceptance, with one participant noting that ‘Each time it is so popular that it is really oversubscribed’. Another strength lay in an efficient institutional structure leveraging the World Bank’s treasury services. However, its viability depends on scale, with one participant stating that ‘you need to have a certain size in order to make sense’. The IFFIm’s model of frontloading resources has been successfully replicated by other financial institutions.

#### Debt2Health

Debt2Health is a debt swap mechanism whereby a creditor government forgives a portion of a debtor country’s any outstanding debt, conditional on the debtor government investing an equivalent amount in local currency in health programmes, typically through the Global Fund. This creates a ‘triangular’ arrangement benefiting all parties: creditors reduce non-performing loans, debtors gain fiscal space while funding health, and global health initiatives receive additional resources. Debt2Health, however, faced challenges related to political cycles, with one participant noting: ‘Each country has its own decision cycles. They have elections... that might delay decision making’. Successful conversions, like Germany’s EUR 50 million swap with Indonesia for tuberculosis programmes, showcased potential. However, reputational risks for both creditor and debtor nations and prolonged negotiations were significant hurdles, requiring a ‘triple win’ for all parties.

#### Micro-levies

Micro-levies are small, mandatory charges on specific transactions or activities, with revenues earmarked for development purposes. The most prominent example in global health is France’s solidarity tax on airline tickets, which has generated hundreds of millions of EUR for Unitaid since 2006. These levies create predictable, long-term funding streams insulated from annual budget negotiations. Micro-levies were noted for creating reciprocal relationships, as seen with France’s solidarity tax. However, their vulnerability to external shocks was starkly illustrated during COVID-19, with one participant noting: ‘During COVID, the entire industry collapsed, eliminating a critical funding flow’. Political resistance to new taxes in high-income countries also limits their feasibility.

### Evolution of risk and resource allocation

A central finding was our participants’ observation of a shift from direct funding to complex risk-sharing. Traditional grants concentrate risk on the donor, while innovative financing mechanisms distribute risk across multiple stakeholders (**Table 2**).

**Table 2:** Risk distribution patterns across financing mechanisms

Mechanism	Risk bearer	Risk distribution	What it means in practice
Traditional grants	Donor	Single-party risk: the donor bears the full financial risk of the grant.	Donor assumes full loss if funds are mismanaged or programmes fail; recipient has limited liability.
Debt2Health	Shared between creditor and debtor	Bilateral risk sharing: both creditor and debtor governments share the financial commitment.	Creditor forgives debt, debtor commits equivalent local currency to health; both bear reputational and financial risks if agreement fails.
Blended finance	Multi-stakeholder	Complex risk distribution: risk is distributed across public, private, and philanthropic partners.	Public/philanthropic capital absorbs first losses to protect private investors; risk is layered and contractually allocated.
IFFIm	Market-based	Widely distributed: risk is effectively distributed among bond investors in capital markets.	Investors bear risk of bond default, backstopped by legally binding donor pledges; risk is priced into bond yields.
Traditional grants	Donor	Single-party risk: the donor bears the full financial risk of the grant.	Donor assumes full loss if funds are mismanaged or programmes fail; recipient has limited liability.

IFFIm – International Finance Facility for Immunization

A World Bank expert contrasted traditional domestic financing (‘public budget… from the government’) with the multi-stakeholder ‘appetite to work together’ required for blended finance. Risk mitigation strategies were multi-layered, focusing on integration with existing systems, strong governance frameworks, and robust monitoring and evaluation. As one participant stressed, ‘It [risk mitigation] requires a very strong monitoring and evaluation system in the country’.

### Transformation of stakeholder relationships

We noted a fundamental increase in coordination complexity from traditional financing to innovative financing. While traditional financing operates through straightforward bilateral channels, innovative financing mechanisms introduce complex, multi-stakeholder networks (**Table 3**).

**Table 3:** Comparative stakeholder patterns in innovative financing

Mechanism	Key stakeholders	Coordination requirements	What coordination entails
Debt2Health	Creditor government, debtor country, global health organisations (such as the Global Fund)	High	Aligning sovereign debt negotiations with health programme objectives; synchronising legal, financial, and health ministries across two countries; multi-year diplomatic engagement
IFFIm	Donor government, bond investors, global health organisations (Gavi), World Bank, implementation agencies	Very high	Coordinating legally binding pledges from multiple donors; synchronising with World Bank treasury for bond issuance; ensuring implementing countries can absorb frontloaded funds
Blended finance	Development banks, global health organisations (such as the Global Fund), government, private sector	Extremely high	Aligning due diligence, audit, and disbursement processes across institutions; negotiating risk-sharing terms; providing technical assistance to prepare investment opportunities; managing differing risk-return expectations

IFFIm – International Finance Facility for Immunization

For example, Debt2Health creates a ‘triangular relationship’, while Blended Finance requires ‘a political will and appetite’ across a complex network. This shift demands new capabilities, with one participant noting: ‘We need to have someone that's to talk about public-private partnership… the thing is that we need to have the right level of optimisation of the different sources’.

### Synthesis of enablers and barriers for project implementation

Successful implementation of innovative financing hinged on three key factors. The first factor was a clear problem definition. Precise targeting of unmet needs and alignment with country priorities was crucial. One participant explained: ‘We have to really understand what the current reforms are… then we have to find the windows of the opportunity’. The second factor was stakeholder alignment, with early engagement and clear role definition being vital. The credibility of an entity like the Global Fund as a funding, rather than implementing agency was key to attracting donors. The last factor was the robust institutional capacity. Implementation success was heavily dependent on existing institutional frameworks, technical expertise, and monitoring and evaluation systems. Besides this, common barriers included high coordination complexity, significant transaction costs, and concerns over long-term sustainability and political support for innovative financing mechanisms.

## DISCUSSION

This study provides an in-depth, practitioner-based perspective on how innovative financing mechanisms are implemented within major global health organisations, revealing operational realities often absent from technical descriptions of mechanism design. The central finding is that innovative financing mechanisms serve as strategic complements, not replacements, for traditional funding. As one official succinctly stated: ‘It is not magic money… well-developed health systems don't fund their health systems through innovative financing schemes’.

Our findings on the increased coordination complexity and critical role of institutional capacity extend the literature beyond technical analyses of mechanism design [8,9] to highlight the operational realities of implementation. The progression in stakeholder complexity, from bilateral (traditional) to triangular (Debt2Health) to networked (Blended Finance), demands sophisticated coordination and alignment of institutional processes that are often underestimated.

Our findings highlight a fundamental distinction between traditional and innovative financing. Traditional financing offers predictability and relatively low transaction costs within established bilateral relationships, making it suitable for sustained health system strengthening. However, its rigidity and dependence on donor budgets limit its responsiveness to emergent needs or large-scale shocks. Innovative financing, conversely, offers flexibility, risk diversification, and access to new capital pools, as seen with IFFIm’s frontloading or blended finance’s leverage. Yet, as our results demonstrate, this comes at the cost of significantly higher coordination complexity and transaction costs. The key disadvantage is that these mechanisms are not universally applicable; they require sophisticated institutional capacity and specific market or political conditions to succeed. Thus, they are not substitutes, but specialised tools within a broader financing portfolio.

The study also refines the understanding of market-based mechanisms. Contrary to being standalone solutions [10], mechanisms like the IFFIm are most effective when integrated within broader financing frameworks, leveraging the credibility of established institutions like the World Bank. This underscores the importance of adaptive learning and building existing systems, rather than creating parallel structures.

This research offers several critical recommendations for policymakers and global health practitioners. First, it underscores the necessity of achieving a strong context-mechanism fit, as there is no universal innovative financing solution. The selection of any specific mechanism must be carefully tailored to a particular health need, the existing local capacity, and the broader stakeholder landscape to ensure its viability and impact. Second, success demands a dedicated investment in coordination capacity. For complex mechanisms like Blended Finance, effective implementation relies heavily on robust coordination capabilities and strategic relationship management as it does on the technical aspects of financial engineering. Finally, there is a need to strengthen foundational systems, as innovative financing mechanisms cannot compensate for or bypass weak underlying institutions. Bolstering core capacities, particularly in monitoring and evaluation, governance, and financial management, is a fundamental prerequisite for the effective and sustainable use of these innovative tools.

This study has some limitations. The sample size, while providing rich qualitative insights, is small and drawn from a specific set of global health organisations, which may limit the generalisability of findings. The homogeneity of participants, with a majority from the Global Fund and all senior-level professionals from major organisations headquartered in Geneva/Washington, may introduce perspective bias, as viewpoints from recipient governments, smaller NGOs, or the private sector, especially those from Global South, are absent.

## CONCLUSIONS

Innovative financing mechanisms represent an evolution in the toolkit for global health funding, offering valuable approaches for resource mobilisation and efficiency gains. However, their contribution lies in complementing, not replacing, traditional funding. Success is not guaranteed by technical design alone but is heavily dependent on the implementation context, robust institutional capacity, and effective management of complex stakeholder relationships. Future efforts should focus on strategic integration of these mechanisms within strengthened health financing systems, with a clear-eyed view of the operational complexities involved.

## Additional material


Online Supplementary Document

## Data Availability

**Data availability:** No quantitative data were collected during this study. Qualitative data are available upon request from the corresponding author, with its sharing subject to relevant regulations of Duke Kunshan University.
