# Supplementary material for: Implementing innovative financing mechanisms in global health – perspectives of stakeholders from global health organisations: a qualitative study
Source: J Glob Health. 2026 Sep 25;16:04260. doi: 10.7189/jogh.16.04260 (PMC13616276; doi:10.7189/jogh.16.04260)
Supplement: Supplementary file 1 [file jogh-16-04260-s001.pdf]

Supplement to: Ching S, Wamsiedel M, Zhao J. Implementing innovative financing mechanisms in global health – perspectives of stakeholders from global health organisations: a qualitative study. J Glob Health. 2026;16:04260.

Appendix A. Interview Guide

Topic: Exploring the Role of Innovative Finance in Addressing Global Health Challenges

Introduction:

- Introductory information
- Informed consent

Background information:

- To start, could you please provide a brief overview of your position and your duties within your organization?
- Thank you for that overview. Now, I'd like to discuss your personal experience with health financing, especially innovative financing. Could you tell me about any involvement or direct experience you have had in utilizing innovative finance mechanisms to mobilize resources for global health initiatives?

Understanding Innovative Finance:

- Could you please define what you understand by the term 'innovative financing mechanisms'?
- Can you identify and describe some distinct kinds of innovative financing mechanisms that you are familiar with? For example, could you explain mechanisms such as social impact bonds, advance market commitments, and health impact bonds?

Approaches and Practices:

- Can you describe how the organization is currently deploying innovative financing methods to implement their programs in resource-poor settings?

Opportunities and Solutions:

- Can you identify any unmet needs in the application of creative and appropriate financial models to overcome some of the important global health problems, particularly among underserved populations and in response to emerging threats?

- In your opinion, how can innovative financing be made more efficient and cost-effective in delivering health benefits?

Collaboration and Partnerships:

- Can you describe the involvement of public-private partnerships and the contribution of other stakeholders in sourcing resources using innovative financing?

- Could you provide any case studies or insights on how successful collaboration has facilitated the achievement of the global health agenda through innovative financing?

Future Directions and Recommendations:

- What is your perspective on the future trajectory of innovative finance in global health?

- Could you provide any recommendations or suggestions for optimizing the organization’s approach to leveraging innovative finance to address current and future health challenges?

Closing:

- Provide opportunities for any further comments or reflections.

- Ask for any final questions or interests of the participants.

- Check for conducting additional interviews to fulfill the insights gathered from this interview.

- Ask for other potential interviewees and their willingness.
